# Silica Exposure Differentially Modulates Autoimmunity in Lupus Strains and Autoantibody Transgenic Mice

**DOI:** 10.3389/fimmu.2019.02336

**Published:** 2019-10-01

**Authors:** Mary H. Foster, Jeffrey R. Ord, Emma J. Zhao, Anastasiya Birukova, Lanette Fee, Francesca M. Korte, Yohannes G. Asfaw, Victor L. Roggli, Andrew J. Ghio, Robert M. Tighe, Amy G. Clark

**Affiliations:** ^1^Department of Medicine, Duke University Health System, Durham, NC, United States; ^2^Durham VA Medical Center, Durham, NC, United States; ^3^Division of Laboratory Animal Resources, Duke University Medical Center, Durham, NC, United States; ^4^Department of Pathology, Duke University Health System, Durham, NC, United States; ^5^National Health and Environmental Effects Research Laboratory, US Environmental Protection Agency, Chapel Hill, NC, United States

**Keywords:** silica, humoral autoimmunity, B cell tolerance, lupus, autoantibody transgene

## Abstract

Inhalational exposure to crystalline silica is linked to several debilitating systemic autoimmune diseases characterized by a prominent humoral immune component, but the mechanisms by which silica induces autoantibodies is poorly understood. To better understand how silica lung exposure breaks B cell tolerance and unleashes autoreactive B cells, we exposed both wildtype mice of healthy C57BL/6 and lupus-prone BXSB, MRL, and NZB strains and mice carrying an autoantibody transgene on each of these backgrounds to instilled silica or vehicle and monitored lung injury, autoimmunity, and B cell fate. Silica exposure induced lung damage and pulmonary lymphoid aggregates in all strains, including in genetically diverse backgrounds and in autoantibody transgenic models. In wildtype mice strain differences were observed in specificity of autoantibodies and site of enhanced autoantibody production, consistent with genetic modulation of the autoimmune response to silica. The unique autoantibody transgene reporter system permitted the *in vivo* fate of autoreactive B cells and tolerance mechanisms to be tracked directly, and demonstrated the presence of transgenic B cells and antibody in pulmonary lymphoid aggregates and bronchoalveolar lavage fluid, respectively, as well as in spleen and serum. Nonetheless, B cell enumeration and transgenic antibody quantitation indicated that B cell deletion and anergy were intact in the different genetic backgrounds. Thus, silica exposure sufficient to induce substantial lung immunopathology did not overtly disrupt central B cell tolerance, even when superimposed on autoimmune genetic susceptibility. This suggests that silica exposure subverts tolerance at alternative checkpoints, such as regulatory cells or follicle entry, or requires additional interactions or co-exposures to induce loss of tolerance. This possibility is supported by results of differentiation assays that demonstrated transgenic autoantibodies in supernatants of Toll-like receptor (TLR)7/TLR9-stimulated splenocytes harvested from silica-exposed, but not vehicle-exposed, C57BL/6 mice. This suggests that lung injury induced by silica exposure has systemic effects that subtly alter autoreactive B cell regulation, possibly modulating B cell anergy, and that can be unmasked by superimposed exposure to TLR ligands or other immunostimulants.

## Introduction

Autoimmune diseases afflict 10–20% of the US population, often striking young adults and destroying vital organs. Abnormal activation of self-reactive B cells and T cells precipitates spontaneous immune attack on the body. Current therapies can dampen the immune response but do so non-specifically and risk serious side effects. There is an urgent need for safer treatments, but their development will require a better understanding of underlying disease pathogenesis. Considerable evidence indicates that autoimmune responses originate from interaction of environmental triggers with disease susceptibility genes, but little is known about the cellular or molecular basis of this interaction. In particular there is a paucity of information about the mechanism by which environmental agents lead to loss of autoimmune regulation, the fundamental defect in these diseases.

Inhalational exposure to crystalline silica dust (silicon dioxide) has been convincingly linked to human autoimmunity (1). Silica is an abundant natural mineral used commercially in multiple industrial applications and in professions where grinding processes produce silica dust. In addition, there is silica exposure in numerous occupations with manipulation of crustal sources (e.g., agriculture and mining). Numerous case series, case-control, and other epidemiological studies link silica exposure to systemic lupus erythematosus (SLE), anti-neutrophil cytoplasmic autoantibody (ANCA)-associated vasculitis (AAV), rheumatoid arthritis (RA), and systemic sclerosis (SSC), reviewed in (1–5). These chronic relapsing autoimmune diseases cause considerable disability, have life threatening consequences, and currently afford limited opportunities for treatment.

A common and remarkable feature of each of these autoimmune diseases is a prominent autoantibody (autoAb) component. Each disease has a characteristic profile of circulating autoAbs that serves as a biomarker to facilitate diagnosis, prognostication, and disease monitoring and that informs treatment decisions. The appearance of high affinity autoAbs often precedes clinical disease, suggesting their importance early in the disease process (6). Considerable experimental data indicate that the autoAbs mediate tissue destruction and play a critical role in disease pathogenesis (7). AutoAbs to nuclear and other self-antigens deposit in and damage kidneys or bind to and deplete blood cells in SLE (8–10); IgG reacting with type II collagen, citrullinated proteins, and Ig itself (rheumatoid factor) destroys peripheral joints in RA (11); IgG bound to neutrophil myeloperoxidase and proteinase3 triggers small blood vessel injury in ANCA vasculitis (12, 13); and antibodies to nuclear antigens and cell membrane receptors facilitate skin and organ fibrosis in SSC (14–16). The common feature in these autoimmune diseases is activation of autoreactive B cells that have escaped B cell tolerance to generate autoAbs.

To understand how inhalation of silica dust leads to breach of immune tolerance and induction of humoral autoimmunity, we took advantage of a preclinical model system developed in part to better mirror the outbred human situation. This unique mouse reporter system was previously generated to study gene-environment interactions in SLE. For this purpose, a lupus autoAb was expressed as a transgene (Tg) in the non-autoimmune C57BL/6 (B6) strain as well as in multiple classic lupus strains (MRL, NZB, BXSB) (17–20). Each lupus strain carries a different constellation of lupus susceptibility genes, such that they *collectively* mirror the genetic complexity of human lupus. Moreover, the selected strains develop clinical and immunological features and incorporate genetic susceptibility relevant to multiple silica-linked diseases: MRL mice develop delayed lupus nephritis, whereas their MRL/lpr congenic counterparts develop aggressive kidney disease and RA-like arthritis (21); a subset develop anti-myeloperoxidase (MPO) autoAb similar to those observed in ANCA vasculitis (22). NZB mice develop IFNα-receptor-dependent lupus with delayed nephritis and severe autoAb-mediated autoimmune hemolytic anemia (23, 24). NZB carry major risk alleles for severe nephritis (25). The BXSB strain carries an aberrant macrophage receptor with collagenous structure (MARCO) and develops nephritis that is accelerated in the presence of the Y-chromosome-linked autoimmune acceleration (*Yaa*) locus that includes a TLR7 duplication (26, 27).

In mice of these strains carrying the autoAb Tg, the fate of Tg autoreactive B cells, Ab, and tolerance phenotypes can be tracked and quantified. The IgMa Tg was originally constructed from the dominant Ig heavy chain of an IgG autoAb derived from a nephritic MRL/lpr mouse (28). The index IgG binds to laminin, a multifunctional glycoprotein expressed in basement membranes and a target of autoAbs implicated in SLE, blistering dermatoses, reproductive failure, and other disorders (29–34). A prominent subset of Tg autoAbs also crossreacts with DNA (28), a prototypical target antigen in SLE. In healthy B6 mice, these autoreactive Tg B cells are stringently regulated by central deletion, anergy (a state of functional unresponsiveness), and receptor editing (17, 18), tolerance phenotypes that are readily measured and have been stable for over 1.5 decades of study in our mouse colony. Thus, the autoAb Tg permits reliable tracking of key tolerance checkpoints using a single disease-relevant autoAb. In the studies described herein, the autoAb Tg system is leveraged to study and quantitate effects of silica exposure on B cell tolerance, under the influence of genetically distinct healthy and disease-prone backgrounds.

## Materials and Methods

### Mice

To study wildtype mice, young adult female autoimmune NZB, BXSB, and MRL mice and healthy C57BL/6 (B6) mice were purchased from Jackson Labs and used between 1.6 and 3 months of age. Generation and characterization of the LamH IgMa+ autoAb Tg was previously described (17, 18). The autoAb Tg was crossed onto the B6 strain a minimum of 21 generations and onto NZB/BINJ (NZB), BXSB, and MRL strains between 9 and 21 generations, and carried as a hemizygote (19). Experimental autoAb Tg mice were bred in our colony and included adult male and female mice between 4.3 and 11.7 months of age, with mice of similar age, primarily littermates, assigned to silica and vehicle instillation within a strain. Mice were housed in microisolators in a specific pathogen-free facility with a 14:10-h light/dark cycle. The care and use of all experimental animals were in accordance with institutional guidelines, and all studies and procedures were approved by the local Institutional Animal Care and Use Committees and conform to institutional standards and to the National Institutes of Health guide for the care and use of laboratory animals.

### Silica Administration

Heat sterilized endotoxin-free crystalline silica (Min-U-Sil-5 crystalline silica) was administered once by oropharyngeal instillation as a suspension in sterile phosphate-buffered saline, at 0.2 mg/gm, as described (35, 36). This dose of silica was chosen to ensure that it overcomes pulmonary clearance mechanisms and results in substantial lung delivery and response, including lung inflammation, recruitment of pulmonary lymphocytes, and induction of tertiary lymphoid structures, as observed in our pilot studies and described in investigation into silica-related lung and immune injury using intratracheal or transoral instillations of 5–10 mg silica (37–39). Saline alone was administered as control.

### Tissue and Organ Harvest and Preparation

Wildtype mice were sacrificed at 1, 2, or 3 months after silica instillation for analysis of the whole lung lavage fluid and blood and organ harvest. AutoAb transgenic mice were sacrificed at times indicated in the text. Following CO_2_ euthanasia, blood was collected from the inferior vena cava and serum stored at −20°C. In some mice the descending aorta was transected and organs perfused with saline via the right ventricle. Spleens were removed into culture media. The trachea was exposed and cannulated with PE-60 tubing (Clay Adams, NJ) and lungs lavaged with PBS and bronchoalveolar lavage fluid (BALF) collected (40). The right lung was removed and single cell suspensions for cell culture and flow cytometry obtained by tissue fragmentation in 1 ml RPMI medium using a blade, followed by digestion at 37°C for 40 min after addition of 5 ml solution containing 1 mg/ml collagenase and 0.2 mg/ml DNase 1, with reaction stoppage by addition of cold 120 mM EDTA and cell collection through a 70 μm strainer prior to ACK red cell lysis. The left lung was isolated, inflated to a pressure of 20 cm H_2_O with 10% formalin, removed, and immersed in fixative for immunohistochemistry (IHC).

### Quantitation of Lymphocyte Subsets by Flow Cytometry

For flow cytometry, freshly isolated red blood cell-depleted lung or spleen cell suspensions were Fcgamma Receptor blocked and stained using fluorescence (FL)-labeled Ig, and isotype controls, for CD45 (leukocytes), CD19 (B cells), CD3 (T cells), IgMa (Ig Tg), and IgMb (endogenous Ig) as previously described (19, 20). Data were acquired using FACScalibur or FACSCanto machines (BD Biosciences, San Jose, CA), and data were analyzed using FlowJo software (Ashland, OR).

### BALF Cell Counts and Evaluation of Lung Injury and Tertiary Lymphoid Structures (TLS)

Cells from the BALF were isolated using centrifugation (1,500 rpm, 15 min) and the supernatant was stored at −80°C. The cells underwent red cell lysis (ACK lysis solution) and were counted using a Cellometer K2 (Nexcelom Bioscience, Laurence, MA). Total cell counts were obtained and normalized to the BALF lavage volume. Cells were then immobilized by cytospin and stained with Diff-Quik staining solution to obtain differential counts. Histological analysis was performed on H&E or PAS stained tissue. Lung injury was scored on a 5-point scale that incorporated inflammation, edema, hemorrhage, necrosis, and fibrosis across whole lung sections (41) by an experienced veterinary diagnostician (YA) blinded to study group, using a Nikon photomicroscope and images acquired using an NIS-Elements Nikon camera. To identify deposited silica particles, lung H&E sections were examined with a polarizing attachment as previously described (42). For lung direct IF and quantitation of lymphoid structures, 10% formalin inflated/fixed whole lungs were oriented similarly in cassettes for paraffin embedding, and sectioned (5 μm), with one full-size section from within the first 250 μm of tissue used for counting. This was based on a pilot study examining silica-exposed lungs (*n* = 3) at multiple (5) depths through the lung, which showed that while the average % lung area containing TLS and TLS composition (B/T cell ratios) were similar at all depths, the overall lung section size decreased after a depth of 250 μm. Lung sections were deparaffinized, heated in 10 mM citrate buffer (pH 6.0) to expose antigen, and stained with anti-B220 (B cells) and anti-CD3e (T cells) using appropriate blocking buffer, then labeled using species-specific TRITC-(B cells) or FITC-(T cells) labeled secondary Ab, and counterstained with DAPI (nuclei). Mouse spleen sections served as a positive staining control. For quantitation of TLS: whole lung sections were scanned at the Alafi Neuroimaging Core (Washington University, St. Louis, MO) and NDP Viewer software (Hamamatsu) used for data collection. Images were gridded and each block assessed for TLS, which we defined as a group of 10+ adjacent B and/or T cells. Where indicated, perimeter, area, and B/T cell composition of each TLS were recorded using the Freehand annotation tool. Total TLS area is normalized to overall lung area for the entire lung section, measured using the Freehand tool. Slides were scored by an investigator blinded to study group.

### Cell Culture

For autoAb measurement assays, lung and spleen cell preparations were RBC-depleted and cells plated in 48- or 96-well plates containing one million cells/mL in RPMI 1640 medium (Sigma, St. Louis. MO) containing 10% heat inactivated fetal bovine serum (HI-FBS), plus 2 mM additional L-glutamine, 100 U/mL Penicillin-Streptomycin, 1X MEM Non-essential Amino Acids, 10 mM HEPES Buffer, pH 7.6, and 1 mM Sodium Pyruvate (all additives from Gibco, Waltham MA). To test for the capacity of superimposed environmental stimuli (microbial products) to enhance autoAb production by B cells from silica-exposed wildtype mice and to test for defective or reversible anergy in B cells from autoAb Tg mice, a subset of cell cultures were stimulated with either 50 μg/mL lipopolysaccharide (LPS, TLR4 agonist, Sigma) or a combination of 2 μg/mL resiquimod (R848, TLR7 agonist, Sigma) and 1 μg/mL ODN 1668 CpG oligos (CpG, TLR9 agonist, Invivogen, San Diego, CA). Cells were cultured for 7–8 days in 5% CO_2_, 37°C. Collected culture supernatants were stored with 0.01% sodium azide as a preservative at −20°C until assay.

### Ig and AutoAb Quantitation by ELISA

ELISA was used to detect lupus-associated anti-DNA and vasculitis-associated anti-MPO autoAb in wildtype mice, and to detect lupus-associated anti-laminin and anti-DNA autospecificities encoded by the Tg in autoAb Tg mice. Total Ig and Ig Tg (IgMa+) concentration in serum, BALF, and culture supernatants was determined by ELISA, as described (17, 19). To detect autoAb, Immulon 2 HB plates (Thermo Scientific, Waltham, MA) were coated overnight at 4°C with antigen diluted in PBS, including ssDNA prepared by phenol chloroform extraction of calf thymus DNA (Worthington Biochemicals, Lakewood, NJ) (43) at 4.5 or 45 μg/mL, human leukocyte MPO (Sigma Aldrich, St. Louis, MO, or Lee Biosolutions, Maryland Heights, MO) at 0.02 or 0.2 U/mL, laminin from Engelbreth-Holm-Swarm mouse sarcoma (Sigma) at 10 μg/mL in PBS, or with diluent (PBS) only. Plates were blocked for at least 60 min with 3% BSA (Sigma) in PBS, incubated with samples for minimum 60 min, then labeled with goat-anti-mouse Ig-alkaline phosphatase or mouse-anti-mouse-IgMa-biotin-conjugated antibody (BD Pharmingen, San Diego, CA) followed by alkaline-phosphatase conjugated streptavidin (Southern Biotech, Birmingham, AL). Bound Ig were detected with phosphatase substrate (Sigma) and OD405 recorded on Emax microplate reader (Molecular Devices) using SoftMax software. Results for binding to antigen were recorded as OD on antigen minus OD on wells coated with diluent only, after subtraction of OD blank (determined for dilution buffer without Ig). Controls include anti-ssDNA IgG H241 (44), anti-MPO mAb clone CLB-MPO-1/1 (Sigma Aldrich), anti-laminin IgG H50 (45), anti-laminin IgM A10C (28), and anti-laminin/anti-DNA transfectant IgM LamH/Vk8Jk5 (18). Unless otherwise indicated, serum was diluted 1:20 in PBS/0.1% BSA for ELISA assays, and BALF and cell culture supernatants were assayed undiluted.

### Statistical Analysis

Data management and statistical analysis were performed using JMP software (SAS, Cary, NC). Differences between silica- and vehicle-exposed mice within each strain were assessed using Wilcoxon each pair, with *p* < 0.05 considered significant. Differences between silica-exposed mice across all strains were evaluated using Kruskal-Wallis rank sums test and the multiple comparisons Steel-Dwass All Pairs test was used to determine which pairs were significant. To examine the capacity of silica exposure to abrogate deletional tolerance in autoAb Tg mice, sample size determination was performed to estimate the minimum number of animals per group that could significantly differentiate high level deletion (measured as low spleen B cell counts) from a non-deletional phenotype in each strain. Using reference values (mean and standard deviation) from our published data (17–19) that includes experimental groups containing mean 25.9 mice/group (range 11–48), *a priori* power analyses suggested that 3 mice/group could attain statistical significance of *p* < 0.05 with a 90% probability.

## Results

### Lung Injury and Inflammation in Diverse Backgrounds After Silica Exposure

**Figure 1:**
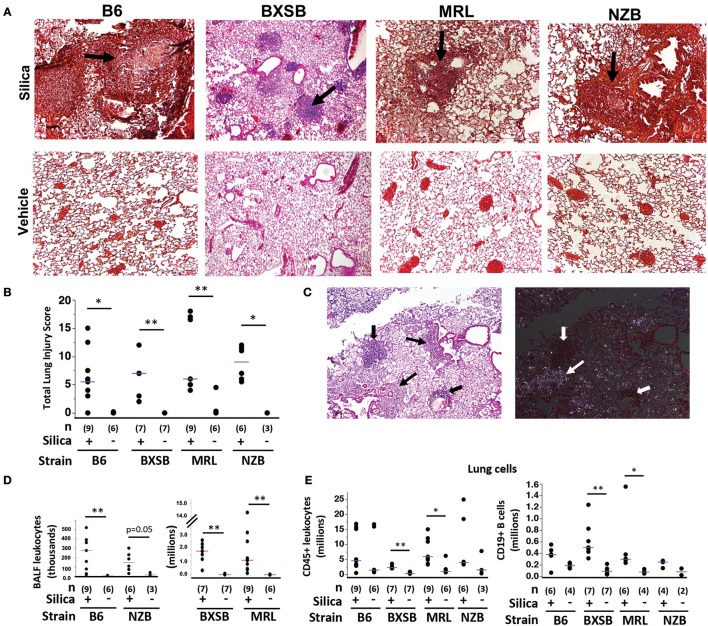
Lung injury and inflammation in diverse wildtype mouse strains after exposure to crystalline silica. **(A)** Representative sections of lung from mice of indicated strain 2 months (MRL) or 3 months (B6, BXSB, NZB) after instillation of silica or vehicle; H&E, original magnification ×40. Arrows indicate granulomas. **(B)** Lung injury composite scores for mice of each strain after exposure to silica or vehicle. **(C)** Localization of silica particles in a representative H&E stained section of lung tissue from an MRL mouse 1 month after silica instillation, viewed by conventional (left) or polarizing (right) light microscopy; thin arrows indicate granulomas, thick arrows indicate lymphoid collections. **(D)** Leukocytes in BAL fluid, counted using Diffquick. **(E)** CD45+ leukocytes (left) and CD45+ CD19+ B cells (right) in whole lung cell isolates of mice after exposure were quantitated by flow cytometry, gated on live cells. For data in scatterplots, lungs were harvested 1, 2, and 3 months post-exposure for each group, except for BXSB for which lungs were harvested at 2 or 3 months post-exposure. Each symbol represents an individual mouse, with tissue harvested at 1, 2, or 3 months after exposure; the median for each group is indicated by the bar; *p < 0.05 and **p < 0.01 for silica- vs. vehicle-exposed mice of same strain, Wilcoxon rank sum test.

To first determine if silica instillation induced lung injury and lymphoid cell accumulation in mice of the different autoimmune genetic susceptibilities, adult wildtype female mice of each background (B6, BXSB, MRL, NZB) were given a single exposure of 0.2 mg/gm (~3–6 mg/mouse) crystalline silica or vehicle (saline) at age 3 months (NZB, B6, MRL) or 1.6 months (BXSB) and analyzed at 1, 2, or 3 months post-exposure. Three time points were chosen for harvest because the effect of this exposure on lung injury and survival in the different backgrounds was unknown. At harvest mice ranged from 3.6 to 6 months old, depending on the month post-exposure. Results showed that all exposed mice survived until the predefined harvest date, at which time mice exposed to silica showed extensive lung injury, whereas vehicle-exposed lungs showed minimal damage (Figures 1A,B). Lung histopathological examination revealed leukocyte infiltration, granuloma formation, alveolar proteinosis, lymphoid collections, edema, and scattered hemorrhage in silica-exposed mice in each strain (Figure 1A). Lung injury was observed at each time point tested, with no correlation between lung injury score and months post-exposure (not shown). Extensive lung injury was already observed by the 1 month time point (Figure S.1). Among all silica-exposed mice, no significant differences were found in lung score between the four strains (Kruskal-Wallis test, ChiSquare = 2.59, *p* = 0.46, *df* = 3). Polarizing light microscopy of lung tissue on H&E stained sections revealed multiple small birefringent particles in lungs of mice exposed to silica, whereas lungs of most mice exposed to vehicle demonstrated only scattered background birefringence. Silica particles were concentrated in pulmonary nodules and granulomas (Figure 1C), and in some animals were scattered throughout lung parenchyma. In contrast, silica particles were generally absent from lymphoid collections.

Silica exposure was associated with leukocytic infiltration of bronchoalveolar space and lungs in each strain. BALF from silica-exposed mice contained significantly more leukocytes than BALF from their vehicle-exposed counterparts (Figure 1D). A Kruskal-Wallis test also showed a significant difference in BALF leukocyte counts between silica-exposed mice of different strains (Table 1). Adjustment for multiple comparisons showed that both BXSB and MRL silica-exposed mice had significantly higher BALF leukocyte counts than did B6 or NZB silica-exposed mice (Steel-Dwass All Pairs method, *p* < 0.05 each pair), whereas there were no significant differences between MRL vs. BXSB or NZB vs. B6 silica-exposed groups. Whereas, macrophages dominated BALF cell composition in vehicle-exposed mice (range 96% to 100% of BALF cells in B6, BXSB and NZB mice, *n* = 16; range 60–99% of BALF cells in MRL, *n* = 6), neutrophils constituted a significantly greater proportion of BALF cells in silica-exposed mice of each strain: % neutrophils, median (IQR), 15% (12.5) for B6, 15% (19) for BXSB, 36% (21.5) for MRL, and 32% (19.5) for NZB, all *p* < 0.05 vs. their vehicle-exposed counterparts. Lymphocytes comprised a small percentage of BALF cells in all strains (not shown).

**Table 1:** Differences between silica-exposed wildtype mice of diverse autoimmune genetic backgrounds.

	**Si-exposed strain**								**Kruskal-Wallis**			**Steel-Dwass all pairs**
	**B6**		**BXSB**		**MRL**		**NZB**		**Chi square**	***df***	***p*-value**	***p*-value**
**Parameter**	**Median**	**IQR^a^**	**Median**	**IQR**	**Median**	**IQR**	**Median**	**IQR**				
**BALF LEUKOCYTE COUNT (THOUSANDS)^b^**												
	275	322	1,772	1124	1,096	2,406	147	185	17.77	3	0.0005	<0.05, BXSB vs. B6 & NZB
												<0.05, MRL vs. B6 & NZB
**LUNG CD45+ LEUKOCYTE COUNT (MILLIONS)^b^**												
	4.6	12.6	2.3	0.7	5.9	7.3	4.0	16.8	11.62	3	0.0088	<0.05, BXSB vs. MRL & NZB
**LUNG TLS COUNT^c^**												
	51	34	29	28	70	46	14.5	20.3	15.55	3	0.0014	<0.05, MRL vs. BXSB
												<0.01, MRL vs. NZB
**LUNG TLS AREA %^c^**												
	0.79	0.84	1.11	1.76	1.47	0.91	0.47	1.23	10.03	3	0.0183	<0.05, MRL vs. B6
**BALF autoAb LEVELS (OD405)^d^**												
α-DNA IgM	0.262	0.255	0.965	0.928	1.386	1.223	0.635	0.340	19.85	3	0.0002	<0.01, B6 vs. MRL
												<0.05, B6 vs. BXSB & NZB
α-DNA IgG	0.058	0.056	0.445	0.661	1.510	1.452	0.273	0.616	20.63	3	0.0001	<0.01, B6 vs. MRL & BXSB
α-MPO IgM	0.000	0.005	0.079	0.094	0.003	0.047	0.008	0.026	12.97	3	0.0047	<0.01, B6 vs. BXSB
**LUNG CELL CULTURE (TLR LIGAND STIMULATED) ANTI-DNA IgG (OD405)^d^**												
TLR7/9	0.011	0.029	–	–	0.312	0.910	0.000	0.013	16.10	2	0.0003	<0.01, MRL vs. B6 & NZB
TLR4	0.000	0.003	–	–	0.100	0.307	0.000	0.014	16.08	2	0.0003	<0.001, MRL vs. B6
												<0.05, MRL vs. NZB
**SERUM ANTI-DNA IgG (OD405)^d^**												
	0.553	0.572	0.278	0.362	2.965	2.536	2.045	1.727	21.35	3	<0.0001	<0.01, MRL vs. B6 & BXSB
												<0.05, NZB vs. BXSB

a *BALF, bronchoalveolar lavage fluid; IQR, interquartile range; df, degrees of freedom; MPO, myeloperoxidase; Si, silica; TLR, toll-like receptor; TLS, tertiary lymphoid structures*.

b *BALF leukocyte counts were determined by Cellometer K2 and lung counts by flow cytometry*.

c *Lung TLS count and % area were quantified on gridded images of scanned whole lung sections stained for B and T cells*.

d *Autoantibodies in undiluted BALF and lung cell culture supernatants and in sera diluted 1:100, except for MRL sera diluted 1:500, were measured using ELISA*.

Flow cytometry of dissociated whole lung revealed that silica-exposed BXSB and MRL mice had significant enrichment for CD45+ leukocytes and CD19+ B cells in their lungs compared to their vehicle-exposed counterparts (Figure 1E). Lung leukocyte counts also differed according to strain among all silica-exposed mice, though in a pattern different from that observed for BALF: silica-exposed BXSB lungs contained significantly fewer leukocytes than lungs of silica-exposed MRL or NZB mice (Table 1). For CD19+ B cell counts, a trend was observed for fewer B cells in silica-exposed NZB vs. BXSB lungs (*p* = 0.05 by Steel-Dwass All Pairs).

### Lung Lymphoid Structures

**Figure 2:**
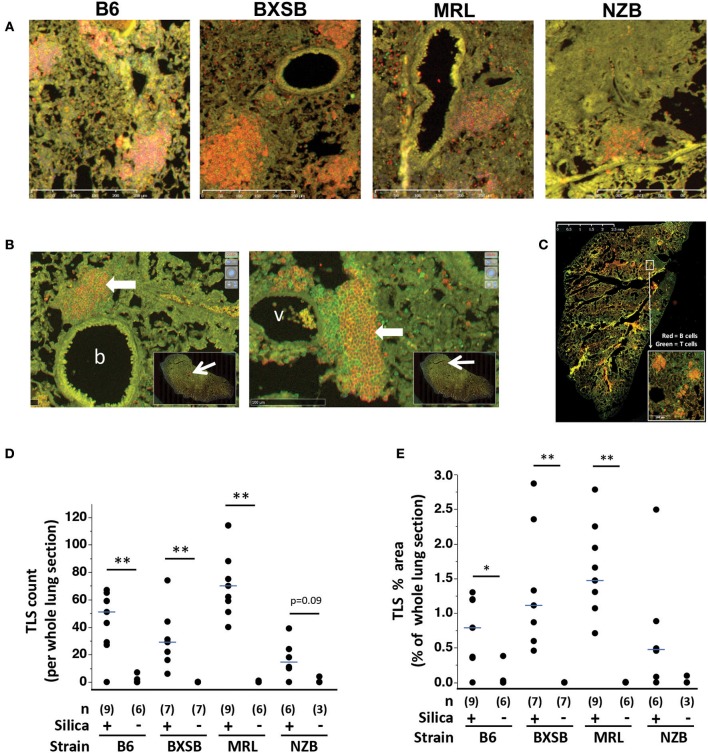
Tertiary lymphoid structures (TLS) in lungs of wildtype mice exposed to silica. **(A)** Representative sections of lung from mice of indicated strain 2 months (MRL) or 3 months (B6, BXSB, NZB) after instillation of silica or vehicle, stained with anti-B220 (B cells, red) and anti-CD3e (T cells, green), original magnification ×100. **(B)** Representative peribronchiolar (left) and perivascular (right) tertiary lymphoid structures (thick arrows), from the lung of an MRL mouse 1 month after silica exposure; b, bronchiole; v, vessel. The insets show the whole lung section, with the area of magnification outlined by the red box at the tip of the arrow. **(C)** Representative whole lung section scanned after staining with fluorescein-conjugated anti-mouse-CD19 (B cells, red) or anti-mouse-CD3e (T cells, green). The inset shows four TLS. TLS were counted and TLS area quantitated across the entire lung section. **(D)** TLS number per whole lung section; and **(E)** TLS area as percentage of area of whole lung section. For scatterplots each symbol represents an individual mouse; the median for each group is indicated by the bar; *p < 0.05 and **p < 0.01 for silica- vs. vehicle-exposed mice of same strain, Wilcoxon rank sum test.

Immunostaining revealed aggregates of B cells and T cells, similar to TLS, scattered in the lungs of wildtype mice exposed to silica (Figure 2A). Many TLS-like aggregates consisted of a B cell-dominant central region with an adjacent or surrounding collection of T cells, and numerous clusters abutted small blood vessels or bronchioles (Figure 2B). Lymphoid aggregates were delineated and quantified on gridded scanned images of whole lung sections (Figure 2C). TLS were identified in lungs of silica-exposed mice of each strain but were rarely observed in their vehicle-exposed counterparts. This difference was statistically significant for B6, BXSB, and MRL mice, measured both as total number (Figure 2D) and as total lymphoid area as percent of total lung area (Figure 2E). For NZB there was a trend toward an increased number of lung TLS in silica- vs. vehicle-exposed mice.

Among silica-exposed mice differences between strains were also observed, with silica-exposed MRL demonstrating a significantly higher TLS count than their BXSB or NZB counterparts and significantly higher TLS percent area than silica-exposed B6 mice (Table 1). Among silica-exposed mice, there was no correlation between TLS count and duration post-exposure (*p* = 0.7966). TLS area did increase with post-exposure duration (Spearman's rho, *p* = 0.0485), primarily in BXSB and MRL mice.

### AutoAb in Lungs and Serum

**Figure 3:**
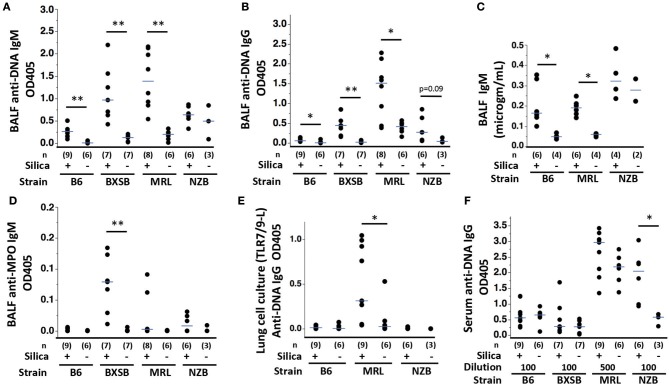
Autoantibody production in wildtype mice of diverse genetic backgrounds exposed to silica. **(A)** Anti-ssDNA IgM; **(B)** Anti-ssDNA IgG; **(C)** IgM concentration; and **(D)** Anti-MPO IgM levels in bronchoalveolar lavage fluid (BALF) from wildtype mice 1–3 months after exposure to silica or vehicle. Autoantibody levels were measured as OD405 for binding to antigen minus binding to diluent-only using undiluted BALF in duplicate. **(E)** Anti-ssDNA IgG levels in supernatants derived from lung cells cultured with a mixture of TLR7 and TLR9 ligands R848 and CpG oligos, from mice of indicated exposure and strain. **(F)** Anti-ssDNA IgG levels in serum diluted 1/100 or 1/500, as indicated. Each symbol represents an individual mouse; the median for each group is indicated by the bar; *p < 0.05 and **p < 0.01 for silica- vs. vehicle-exposed mice of same strain, Wilcoxon rank sum test.

Anti-DNA IgM and IgG autoAb were significantly higher in BALF of silica-exposed mice compared to their vehicle-exposed counterparts for the B6, BXSB, and MRL strains (Figures 3A,B). Silica-exposed B6 and MRL also had higher BALF IgM levels compared to vehicle exposed mice (Figure 3C). Conversely, anti-DNA Ig levels in NZB BALF did not vary by exposure (Figures 3A–C), although there was a trend to higher levels of anti-DNA IgG in BALF of silica-exposed NZB. IgM antibodies to MPO, a major target antigen in ANCA vasculitis, were significantly elevated in BALF of silica- compared to vehicle-exposed BXSB mice (Figure 3D). Low levels of anti-MPO IgG were detected in BALF of several silica-exposed mice of other strains; however, overall levels did not exceed those in their vehicle-exposed counterparts. Among all silica-exposed mice, B6 had significantly lower levels of BALF autoAb than other strains, including less anti-DNA IgM than silica-exposed mice in each of the lupus strains, less anti-DNA IgG than silica-exposed MRL and BXSB, and less anti-MPO than exposed BXSB (Table 1).

Isolated lung cells from exposed B6, MRL and NZB mice were cultured with or without ligands to TLRs to assess silica-related responsivity, with and without the additional environmental (TLR) stimulus. Ligand to TLR4 or a mixture of ligands to TLR7 and TLR9 were used, based on our prior experience demonstrating additive effect of TLR7 and 9 ligands in unmasking reversible anergy in NZB lupus (20). Both TLR solutions induced anti-DNA IgM, including from lung cells of both silica- and vehicle-exposed mice; the highest levels of anti-DNA IgM were induced by TLR7/9 ligand combination (not shown). Quantitation of anti-DNA IgG revealed significantly higher levels in supernatants of lung cells harvested from silica-exposed MRL mice and co-cultured with TLR7/9 ligands compared to similarly cultured lung cells from vehicle-exposed MRL mice (Figure 3E). In contrast, recovery of TLR7/9-induced IgG anti-DNA was not increased in lung cultures from silica- vs. vehicle-exposed B6 or NZB mice (Figure 3E). Among all silica-exposed mice (B6, MRL, and NZB), anti-DNA IgG production by TLR7/9-stimulated lung cells was significantly higher for MRL compared to B6 or NZB (Table 1). For MRL mice, there were trends toward higher spontaneous (in the absence of TLR ligand additive) and TLR4 ligand-induced IgG anti-DNA levels in cultures of lung cells derived from silica- compared to vehicle-exposed mice (*p* = 0.0972 and *p* = 0.0987, respectively, not shown). Among all silica-exposed mice, levels of TLR4-stimulated anti-DNA IgG were significantly higher for MRL compared to B6 and NZB (Table 1).

Elevated levels of IgM anti-MPO were detected in supernatants of TLR7/TLR9 stimulated cultured lung cells from 2 of 9 silica-exposed, vs. 0 of 6 vehicle-exposed, B6 mice; however, overall anti-MPO autoAb levels did not significantly exceed those in vehicle-exposed counterparts (not shown). Ig reactive with alpha3(IV)NC1 collagen, the target antigen in Goodpasture's Disease/anti-glomerular basement membrane nephritis, diseases with serologic and clinical overlap with ANCA vasculitis, were detected in several supernatants of TLR7/TLR9-stimulated cultured lung cells from MRL and B6 mice, including cells from vehicle- as well as silica-exposed subjects (not shown).

Mice of all strains had detectable serum IgM and IgG anti-ssDNA. Anti-DNA IgM and IgG levels did not differ significantly between silica- and vehicle-exposed mice at tested dilutions with the exception of NZB anti-DNA IgG levels, which were significantly higher in silica-exposed mice (Figure 3F). Among silica-exposed mice, serum anti-DNA IgG levels in MRL significantly exceeded those in B6 and BXSB (and likely exceeded those in NZB, noting that MRL serum was tested at 5-fold greater dilution) (Figure 3F and Table 1); levels in NZB serum also exceeded those in BXSB (Table 1).

A few mice of different strains had detectable serum anti-MPO Ig, without a discernable difference based on exposure. Splenocytes from all strains and from mice exposed both to silica and vehicle produced IgM anti-ssDNA in culture, with increased levels after TLR ligand stimulation. TLR4 ligand induced the highest level of IgG anti-DNA from MRL splenocytes, although induced levels did not differ significantly between exposure groups (not shown). BXSB splenocytes uniquely produced high levels of IgM anti-MPO after TLR ligand stimulation; however, levels did not vary based on exposure (not shown).

### Silica Exposure in AutoAb Tg Mice

A major goal of these studies was to directly assess the impact of silica instillation on autoreactive B cell fate and pathogenic autoAb production. For this purpose, we took advantage of an established autoAb Tg reporter system in which B6, NZB, MRL, and BXSB mice carry the LamH IgMa autoAb Tg (17, 18). LamH encodes a dominant Ig heavy chain that generates Ig reactive with laminin and DNA. Expression of the autoAb Tg enriches for B cells with this autospecificity that can be readily tracked using allotypic or idiotypic markers. This model permits measurement of major tolerance mechanisms: deletion is quantitated using spleen B cell counts; anergy can be assessed in part by autoAb production and B cell response to TLR4 stimulation; and receptor inclusion or editing can be measured by expression of endogenous Ig chains. This overcomes some limitations of dissecting mechanisms in wildtype mice with highly diverse polyclonal B cell populations and specificities in which tracking the fate of individual B cells is difficult.

For these experiments, adult autoAb Tg mice were given a single exposure of 0.2 mg/gm (~3–6 mg/mouse) crystalline silica or vehicle (saline) and analyzed after 3.5–7 weeks. AutoAb Tg mice were age-matched to silica vs. vehicle exposure within each strain using littermates (Tg+ mice are not age-matched across strains because between-strain comparison was not a primary goal of this study that measures effects of silica exposure on B cell tolerance mechanisms). Ages are reported for individual autoAb Tg mice in Table S.1. Older adult mice were included, as we rationalized that breach of tolerance, if present, may be more likely detected in older individuals. All autoAb Tg mice were exposed on the same date, and within each autoAb Tg strain mice in the two exposure groups were harvested on the same date. B6-Tg, BXSB-Tg, and MRL-Tg mice were harvested 1.5–1.75 months after silica exposure, a time point based on results in wildtype mice, in which extensive lung inflammation and TLS were already observed by 1 month after silica instillation. A similar post-exposure duration was planned for NZB-Tg mice; however due to weight loss and death of 3 NZB-Tg mice within 3 weeks post-exposure, the remaining NZB-Tg were harvested at this time point and are included in this report.

Lung injury was observed in all autoAb Tg mice exposed to silica, and included inflammation, necrosis, fibrosis, alveolar proteinosis, and edema, whereas vehicle-exposed lungs showed no or minimal injury (Figure S.2A). Immunostaining revealed aggregates of B and T cells consistent with TLS scattered in the lungs of the silica-exposed subset in each strain (Figures S.2B,C). Overall TLS counts per whole lung section (58.7 ± 29.8, mean ± SD, *n* = 12) in the silica-exposed autoAb Tg mice were comparable to TLS counts in wildtype mice (45.1 ± 30.1, *n* = 31). Similar to the case in wildtype mice, numerous lymphoid clusters were located adjacent to small blood vessels or bronchioles (Figure S.2D). Semiquantitative assessment of TLS composition identified a relatively low proportion of B cells within lymphoid clusters in autoAb Tg mice on the MRL and NZB backgrounds (Figure S.2E). Flow cytometric analysis confirmed that B cells expressing the Tg+ allotype IgMa were represented among lung infiltrating B cells (Figure S.2F).

### Tolerance Phenotypes in Silica-Exposed AutoAb Tg Mice

**Figure 4:**
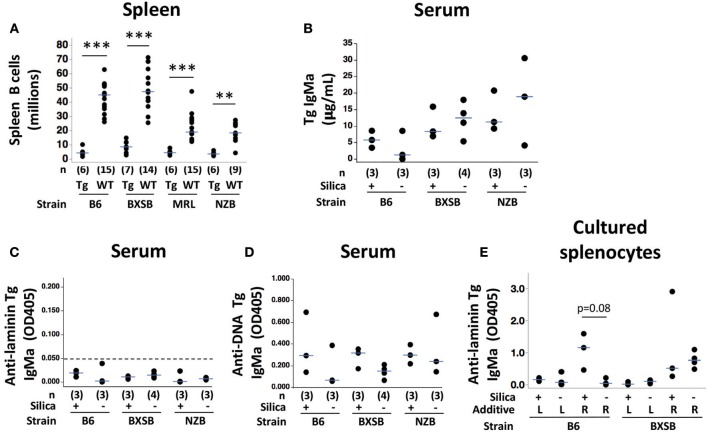
Autoreactive B cell regulation in autoantibody transgenic (autoAb Tg) mice with or without exposure to crystalline silica. **(A)** Spleen CD19+ B cell numbers as determined by flow cytometric analysis in autoAb Tg and WT mice. Within an indicated strain, silica and vehicle-exposed mice are pooled for each genotype; **p < 0.01, ***p < 0.001 for autoAb Tg vs. wildtype mice of the same strain, Wilcoxon rank sum test. **(B)** Serum IgMa (Tg) concentration, determined using a standard curve; **(C)** serum anti-laminin Tg autoAb, with cutoff for laminin-binding positivity at an OD of 0.050 indicated by the dashed line; and **(D)** serum anti-ssDNA Tg autoAb. Concurrent mean OD405 for positive control Tg-expressing IgM monoclonal antibodies are 0.208 for anti-laminin Ig A10C and 0.352 for anti-DNA Ig LamH/Vk8Jk5 (not shown). Antigen binding was tested in serum at 1/20 dilution in duplicate, measured as OD405 on antigen after subtraction of OD405 for binding on diluent-coated wells. **(E)** Level of anti-laminin Tg IgMa autoAb in supernatants of spleen cells from exposed mice stimulated 7–8 days with indicated additive: L, lipopolysaccharide (TLR4 ligand); R, R848 (TLR7 ligand) in combination with CpG oligos (TLR9 ligand). Undiluted supernatants were tested in duplicate; *n* = 3 mice/strain. Concurrent OD405 for positive control monoclonal antibody was 0.974 for anti-laminin H50 IgG.

Despite evidence of extensive lung injury and TLS formation in silica-exposed adult autoAb Tg mice across a range of ages and autoimmune backgrounds, overt breach of B cell tolerance was not detected in any mouse. The total number of splenic B cells was very low in all autoAb Tg mice studied (5.7 ± 3.4 million, mean ± SD, *n* = 25), regardless of exposure or background strain (values for individual mice by strain are shown in Figure 4A). In each of the four strains, the mean number of B cells per spleen in Tg+ mice was very similar to that previously reported in our colonies. Spleen B cell counts (millions, mean ± SD) by autoAb Tg strain were 4.9 ± 2.8 for B6, 8.8 ± 4.3 for BXSB, 4.9 ± 1.7 for MRL, and 3.9 ± 1.3 for NZB. This compares to historical mean counts of 6.0, 10.6, 5.6, and 3.6 million, respectively, for these autoAb Tg strains (17, 19). We observed similar consistency in spleen B cell number among different cohorts of B6 Tg+ mice in our colonies evaluated many years apart (17–19, 46). Historically the spleen B cell count in unmanipulated autoAb Tg+ mice represented a 76–89% reduction in the total number of splenic B cells compared to non-Tg (wildtype) counterparts, in which average B cell counts ranged from 18.2 million in NZB to 60.6 million in BXSB (19). Although age-matched non-Tg littermates were not evaluated simultaneously in the current study, preventing direct comparison of autoAb Tg+ and non-Tg spleen B cell counts, spleen B cell counts of the commercially-acquired adult wildtype mice exposed to silica or vehicle and described in the current study (*n* = 53) were comparable to historical values (Figure 4A). Within each strain, spleen B cell counts were highly significantly lower in autoAb Tg mice compared to wildtype mice for B6, BXSB and MRL, and significant with *p* = 0.0027 for NZB (Figure 4A). For all silica-exposed autoAb Tg mice, mean spleen B cell count was 5.3 ± 3.5 million (*n* = 12). Collectively, the findings support the notion that a large number of B cells in the autoAb Tg+ mice undergo deletional tolerance and that exposure to silica and its associated lung inflammation does not promote a major breach in this central regulation.

The number of splenic B cells did not differ between silica- and vehicle-exposed groups in any autoAb Tg strain (Figure S.3A), nor did the number of splenic B cells in silica-exposed autoAb Tg mice differ between strains (Kruskal-Wallis test, ChiSquare = 3.62, *p* = 0.3061, *df* = 3). The number of mice per individual exposure group is small, however, and a larger number of subjects will be needed to detect lesser, partial, or subtle defects in deletional regulation, if present.

The functional status of the residual Tg B cells was assessed by quantitating Ig production, an indicator of B cell activation and differentiation. For this purpose, levels of serum Tg-encoded IgM and autoAbs were measured in B6-Tg, BXSB-Tg, and NZB-Tg mice, in which Tg allotype IgMa is readily distinguished from endogenous IgMb (levels are not reported for MRL mice, in which endogenous IgM is j-allotype that crossreacts with IgMa, such that differentiation of Tg from endogenous Ig is not possible using anti-allotype reagents). Serum IgMa concentrations ranged from 1.2 to 30.5 μg/mL (Figure 4B), similar to the low levels we previously observed in LamH autoAb Tg mice and 10- to 100-fold lower than normal serum IgM concentrations in wildtype mice (47). Serum levels of Tg anti-laminin autoAb were very low (Figure 4C), consistent with ongoing regulation of the residual Tg anti-laminin B cells that escaped deletion. Anti-ssDNA autoAb encoded by Tg IgMa were detected in serum, indicating that at least a subset of residual Tg-encoded B cells are activated *in vivo* (Figure 4D). Low levels of Tg IgMa were detected in BALF (range 0.001–0.178 μg/mL), with a trend toward higher levels in silica compared to vehicle-exposed mice in B6 and BXSB strains (not shown). Whereas, trace amounts of anti-DNA IgMa were detected in BALF from several mice, no anti-laminin IgMa was detected. Collectively, these findings are consistent with preservation of anergy in residual (non-deleted) anti-laminin Tg B cells, despite exposure to silica.

Another hallmark of anergy in autoAb Tg models is failure of TLR4 ligand LPS to induce Tg autoAb from cultured splenic B cells. In the current experiments sufficient spleen cells were available from B6-Tg and BXSB-Tg mice to assay effects of TLR ligand stimulation. Only low levels of Tg anti-laminin IgMa were induced regardless of silica exposure of the donor autoAb Tg mouse (Figure 4E). This finding supports preservation of anergy in the residual Tg B cells. Surprisingly, stimulation with a combination of TLR7/TLR9 ligands induced substantial levels of Tg anti-laminin autoAb. In B6-Tg mice, there was a trend for greater anti-laminin Tg Ig production by TLR7/9-stimulated B cells from silica- compared to vehicle-exposed mice, despite plating of similar numbers of B cells (Figure 4E). A similar trend was seen for TLR7/9 induction of Tg anti-DNA autoAb from silica-exposed B6 B cells (Figure S.4).

There is also evidence that the Tg B cells are regulated in part by receptor editing, as previously described for this model (18). Flow cytometric analysis of lung cells using allotype-specific anti-sera confirmed the presence of IgMa+ Tg B cells among lung leukocytes in B6-Tg, BXSB-Tg, and NZB-Tg strains (Figure S.2F). In all autoAb Tg mice the percent of lung CD45+CD19+ B cells that are IgMa+ was low (range 3.2–38.9%), a finding not accounted for by endogenous IgMb staining (Figure S.2F). This suggests downregulation of surface IgM, a feature frequently seen in B cell anergy. In the spleen, surface Tg (IgMa) expression on CD19+ B cells varied inversely with that of endogenous IgMb (Figure S.3B), with lowest IgMa levels in B6-Tg mice and only a low frequency of double positive cells (range 0.7–5.7% of CD19+ cells, not shown). This suggests that editing including heavy chain allelic inclusion is operative, particularly in the B6 strain.

## Discussion

Results from silica exposure in mice of genetically diverse backgrounds, including non-autoimmune B6 and autoimmune-prone BXSB, MRL, and NZB strains, substantiate silica's universal capacity to induce pulmonary injury and lymphoid aggregates and demonstrate strain-specific effects on humoral autoimmunity and B cell tolerance. We detected strain differences in autoAb specificity and in the site of enhanced autoAb production, consistent with genetic modulation of the autoimmune response to silica. Using an autoAb Tg reporter system to track the *in vivo* fate of autoreactive Tg B cells, we were able to measure several tolerance mechanisms within the different strains after exposure to a silica dose capable of inducing severe lung injury and TLS in each strain. We observed gross preservation of autoreactive B cell regulation in each autoAb Tg strain: spleen B cells remained markedly depleted in all Tg strains, consistent with intact central immune tolerance, and the residual population of autoAb Tg B cells contributed only low to modest levels of serum Tg IgMa and minimal Tg anti-laminin Ig, regardless of exposure. These tolerance phenotypes mirror those previously reported for unmanipulated mice bearing this autoAb Tg (17, 18), and indicate that silica exposure alone does not overtly disrupt central B cell tolerance. This suggests that silica exposure subverts tolerance at alternative checkpoints, such as regulatory cells or follicle entry, or requires additional interactions or co-exposures to induce loss of tolerance.

It is interesting to note that autoAb Tg B cells localize to lungs of silica-exposed mice in each strain, as demonstrated by flow cytometry and immunohistochemistry, and aggregate in TLS-like clusters. The potential of these B cells to increase local production of autoAb after silica exposure and resulting lung inflammation in B6 and BXSB mice is suggested by the trend toward increased levels of Tg Ig in BALF of silica-exposed mice in these strains; nonetheless, BALF levels of Tg IgMa remain low and anti-DNA and anti-laminin Ig levels are negligible in this setting.

### Systemic Effects of Silica on Autoimmune Regulation

An unexpected finding was the induction of substantial levels of anti-laminin autoAb Tg by splenocytes harvested from B6 mice exposed to silica and co-cultured with ligands to intracellular TLR7 and TLR9. The capacity of the TLR ligands to elicit anti-laminin Ig from Tg B cells from non-autoimmune B6 mice is surprising, because on this background the autoAb Tg is stringently regulated by deletion, editing, and anergy, a tolerance phenotype that has been remarkably stable for over 1.5 decades of study in our mouse colony. Tg anti-laminin Ig have rarely been detected in or recovered from B6 autoAb Tg mice (17, 18). In the current study, the induction of anti-laminin Tg Ig is not observed with splenocytes from vehicle-exposed autoAb Tg B6 mice, and the difference in autoAb production is not explained by differences in B cell numbers, which are equal in the two groups of B6 mice. This suggests that silica-exposed B6 mice have a unique population of autoreactive splenic B cells that can be activated by TLR7/9 ligands and that are not present in their vehicle-exposed counterparts. This supports the notion that the injury induced by silica inhalation has systemic, not just local, effects that subtly alter autoreactive B cell regulation and that can be unmasked by superimposed exposure to TLR ligands. Some potential differences may have been missed in the current study because of the low sample size; these can be tested in a larger follow up study.

A plausible explanation for the subtle deregulated autoimmunity revealed in the spleen B cell differentiation assays is that the systemic immune and cytokine milieu created by silica-induced lung inflammation modulates B cell anergy and promotes generation of a population of reversibly anergic B cells. The phenotype observed in silica-exposed B6 splenic B cells is reminiscent of the reversible anergy previously observed among NZB autoAb Tg B cells, which respond to TLR ligands with production of autoAb Tg, in contrast to anergic B cells in other strains (19). As previously reported, the NZB reversible anergy phenotype is revealed by both TLR4 and TLR7/9 stimulation and is also observed in B cells from autoAb Tg NZB F1 progeny (20), but not in anergic B cells from autoAb Tg B6 or BXSB mice (19). Whereas, the NZB phenotype is genetically-determined and likely cell intrinsic, the cause of development of a TLR7/9 reversible anergy in B cells from silica-exposed B6 mice is unclear and may well involve a distinct mechanism.

Study of models of B cell anergy in non**-**autoimmune-prone mice suggest that maintenance of anergy in healthy cells centers on mechanisms that block TLR ligands from inducing autoAb secretion (48–51). Known mechanisms involve altered MAPK activation or function, in some cases accompanied by altered B cell receptor and/or TLR trafficking and exclusion from late endosomes (site of activation of endosomal TLRs). Increased basal p-ERK, blocked p-ERK nuclear import, and decreased JNK activation have been described. These mechanisms are consistent with central roles of Ras/MAPK pathways in B cell tolerance, TLR signaling, and activation of Blimp-1 (52, 53), the transcriptional regulator that induces plasma cell differentiation and Ig secretion after BCR/TLR stimulation. In this regard, MAPK activation is described in human lung epithelial cells exposed to crystalline silica in culture (54); however, direct exposure seems an unlikely cause of silica-modulated anergy in splenic B cells. Rather exposure of developing or anergic B cells to the sustained systemic proinflammatory milieu created during non-resolving lung inflammation is a more likely culprit. Potential candidates to mediate a breach of B cell anergy include endogenous TLR ligands—such as extracellular matrix components or high-mobility group box 1 (HMGB1) protein—released from injured lung tissue or cells and that can reach high systemic levels in patients with chronic inflammation (55). In this regard, TLR signaling has been shown to reverse anergy in human autoreactive chronic lymphocytic leukemia B cells (56).

It is possible that silica exposure and lung injury interfere with B cell regulation by other mechanisms. A systemic effect on the fate of developing B cells in the bone marrow is plausible. Proinflammatory factors, including interferon and tumor necrosis factor (TNF), have a profound impact on and direct reprogramming in hematopoietic stem cell fate (57). It is thus notable that TNF-α is elevated in plasma of silica-exposed (NZBxNZW)F1 lupus mice (58). Subsequent modulation of B cell signaling thresholds could alter tolerance induction and deletion and allow a small subset of normally censored autoreactive B cells to escape to the periphery. Alternatively, the pulmonary and systemic proinflammatory milieu induced by silica exposure could interfere with extrinsic B cell regulation. This has been observed for some anergic B cells, in which Blimp-1 induction and autoAb secretion is suppressed by dendritic- or macrophage-derived suppressive factors, such as IL6, sCD40L, or TNFα (59–61). This is possible in the assays reported here, which used spleen cell, not isolated B cell, cultures. It is plausible that in some settings silica-induced innate cell activation shifts the balance of secreted factors to release cytokine-mediated B cell suppression. This suggests a milieu distinct from that described in silicosis, in which IL6 and TNF-α levels are typically increased (62).

The relevance of these findings to human autoimmunity will require further study. Epidemiological studies link autoimmunity to occupational exposure to inhaled silica dust. For practical reasons, we and others have modeled silica pulmonary exposure using bolus instillations rather than chronic dust inhalation. The dose administered in our studies (3–6 mg/mouse) is similar to the cumulative dose of 4 mg/mouse, administered by 4 weekly intranasal instillations, used by Bates and colleagues (58), who calculated that 4 mg was the mouse equivalent of one-half of a human lifetime occupational exposure based on recommended exposure limits. This and similar bolus exposures lead to lung injury, TLS, and autoantibody production in mice, suggesting that the protocols provide useful models to study mechanisms of silica-induced autoimmunity. Ultimately, however, relevance of mechanisms to silica-induced injury in humans will require study of alternative exposures, inhalation in particular, and optimized models, including human lung and immune organoids and animals with humanized immune systems, genes, and cytokines.

### Local Autoimmune Regulation After Silica Instillation

The role of the induced pulmonary TLS-like structures in modulating B cell autoimmunity in these strains remains unclear and will require further study, as the role may vary in wildtype vs. autoAb Tg strains. Silica-induced pulmonary TLS in mice is well-described (39, 58, 63–65), and the enhanced humoral autoimmunity described here parallels the findings of Brown, Bates, and Mayeux in lupus-prone New Zealand Mixed 2410 and (NZBxNZW)F1 (BWF1) strains and diversity outbred mice (39, 58, 64). TLS are functional and can provide a microenvironment for local B cell and helper T cell interactions, foreign antigen-driven immune responses, and local antibody production (66–69). TLS may also promote autoimmunity. Autoreactive B cells were detected in ectopic germinal centers of salivary gland from patients with Sjogren's syndrome, suggesting that TLS lack normal regulatory checkpoints, such as follicular exclusion found in secondary lymphoid organs (70). Moreover, synovial TLS dissected from RA patients' joints produce human anti-citrullinated-protein IgG when transplanted into scid mice (71, 72).

There is evidence for a role for lung TLS in promoting local autoAb production in the wildtype mice in the present study, although with strain-specific differences. Silica exposure led to significantly elevated levels of anti-DNA Ig in BALF in all strains except NZB, in which it trended toward significance. Levels of anti-DNA Ig in silica-exposed B6 mouse BALF were nonetheless quite low, relative to their autoimmune counterparts (Figures 3A,B and Table 1). Conversely, levels of BALF anti-DNA Ig in silica-exposed MRL mice significantly exceeded those in other silica-exposed strains. Whereas, it is possible that these high BALF levels in MRL derive from elevated serum anti-DNA levels, the recovery of significantly more anti-DNA IgG from TLR-stimulated lung cells of silica-exposed MRL mice compared to other strains (Figure 3E) suggests that abundant IgG is produced locally.

Anti-MPO Ig were also detected in BALF of silica-exposed mice, although with a different strain distribution. Levels of anti-MPO Ig in BALF from BXSB mice significantly exceeded levels in other silica-exposed strains. This strain restriction was unexpected, in that we initially screened for autoAb to MPO, a major autoAb specificity linked to ANCA vasculitis, both because of the association of silica exposure with ANCA vasculitis in humans and because of reports of detection of anti-MPO Ig in subsets of MRL/lpr mice (22) as well as in the derivative autoimmune SCG/Kj strain (73, 74). These results suggest that BXSB as well as MRL genes contribute to anti-MPO production in SCG/Kj mice. We did not observe anti-MPO Ig in silica-exposed B6 mice, a strain in which B cell responses to self-MPO are difficult to induce in the absence of genetic manipulation of antigen expression (75).

The role of TLS in autoAb Tg mice is likely more complicated and more challenging to dissect. The autoAb Tg is highly useful for tracking B cells of known autospecificity and tolerance mechanisms that control them, considerations that guided the choice of model for this study. However, introduction of an Ig Tg by design generates a relatively homogeneous B cell population, with elimination of much of the endogenous B cell population by allelic exclusion. An autoAb Tg further skews the repertoire, particularly if regulation markedly depletes and inactivates B cells, as is the case with the autoAb Tg under study here. B cell repertoire restriction also impacts T cell numbers, immunity, and subset distribution. Because functional, activated B cells and T cells may be critical for initiation, organization, and maintenance of TLS (76), this process may be disrupted in autoAb Tg mice. Moreover, the influence of anergic or regulatory B cells on TLS biology is less clear. Nonetheless it is of note that our silica-exposed autoAb Tg mice develop multiple TLS-like structures in their lungs.

In summary, our findings collectively suggest that silica exposure and subsequent sustained lung inflammation lead to strain-specific modulation of humoral autoimmunity that includes subtle effects on B cell tolerance. The type of autoAb induced varies by autoimmune genetic susceptibility and by site of autoAb production, as revealed by local production of anti-MPO autoAb in silica-exposed BXSB lung. However, silica exposure alone is insufficient to overtly disrupt B cell deletional or anergric tolerance, even at doses capable of inducing striking lung injury and TLS formation in multiple autoimmune backgrounds. Evidence is provided that superimposed exposure to TLR ligands may collaborate with silica-induced immune aberrations to promote autoAb production. Future studies can define the local and systemic immune phenotypes induced by silica exposure, including the pulmonary immune response in autoAb Tg mice, and further dissect the cellular and molecular basis of aberrant tolerance after silica exposure and role of synergistic environmental susceptibility, the results of which may inform design of inhaled or systemic immunotherapies.

## Data Availability Statement

All datasets generated for this study are included in the manuscript/Supplementary Files.

## Ethics Statement

This study was carried out in accordance with the recommendations of the National Institutes of Health guide for the care and use of laboratory animals. The protocol was approved by the Institutional Animal Care and Use Committees of Duke University and the DVAMC.

## Author Contributions

MF, RT, AG, and AC contributed to conception and design of the study. JO, EZ, AB, FK, LF, and AC performed the experiments. YA scored the lung histopathology. VR reviewed and documented the lung pathology and silica deposition. MF, JO, LF, and AC performed the statistical analyses. MF wrote the first and revision drafts of the manuscript. All authors contributed to manuscript revision, read, and approved the submitted version.

### Conflict of Interest

The authors declare that the research was conducted in the absence of any commercial or financial relationships that could be construed as a potential conflict of interest.

## Abbreviations

ANCA: anti-neutrophil cytoplasmic autoantibody
AAV: ANCA-associated vasculitis
autoAb: autoantibody
BALF: bronchoalveolar lavage fluid
MPO: myeloperoxidase
RA: rheumatoid arthritis
SLE: systemic lupus erythematosus
SSC: systemic sclerosis
Tg: transgene/transgenic
TLS: tertiary lymphoid structures.

## References

[1] Miller FW Alfredsson L Costenbader KH Kamen DL Nelson LM Norris JM. Epidemiology of environmental exposures and human autoimmune diseases: findings from a National Institute of Environmental Health Sciences Expert Panel Workshop. J Autoimmun. (2012) 39:259–71. 10.1016/j.jaut.2012.05.002 22739348 PMC3496812

[2] Parks CG Conrad K Cooper GS. Occupational exposure to crystalline silica and autoimmune disease. Environ Health Perspect. (1999) 107:793–802. 10.1289/ehp.99107s5793 10970168 PMC1566238

[3] de Lind van Wijngaarden RA van Rijn L Hagen EC Watts RA Gregorini G Tervaert JW. Hypotheses on the etiology of antineutrophil cytoplasmic autoantibody associated vasculitis: the cause is hidden, but the result is known. Clin J Am Soc Nephrol. (2008) 3:237–52. 10.2215/CJN.03550807 18077783

[4] Chen M Kallenberg CG. The environment, geoepidemiology and ANCA-associated vasculitides. Autoimmun Rev. (2010) 9:A293–8. 10.1016/j.autrev.2009.10.008 19892038

[5] Dospinescu P Jones GT Basu N. Environmental risk factors in systemic sclerosis. Curr Opin Rheumatol. (2013) 25:179–83. 10.1097/BOR.0b013e32835cfc2d 23287382

[6] Rawlings DJ Metzler G Wray-Dutra M Jackson SW. Altered B cell signalling in autoimmunity. Nat Rev Immunol. (2017) 17:421–36. 10.1038/nri.2017.24 28393923 PMC5523822

[7] Ludwig RJ Vanhoorelbeke K Leypoldt F Kaya Z Bieber K McLachlan SM. Mechanisms of autoantibody-induced pathology. Front Immunol. (2017) 8:603. 10.3389/fimmu.2017.00603 28620373 PMC5449453

[8] Mannik M Merrill CE Stamps LD Wener MH. Multiple autoantibodies form the glomerular immune deposits in patients with systemic lupus erythematosus. J Rheumatol. (2003) 30:1495–504. Available online at: http://www.jrheum.org/content/30/71495 12858447

[9] Bonanni A Vaglio A Bruschi M Sinico RA Cavagna L Moroni G. Multi-antibody composition in lupus nephritis: isotype and antigen specificity make the difference. Autoimmun Rev. (2015) 14:692–702. 10.1016/j.autrev.2015.04.004 25888464

[10] Skare T Picelli L Dos Santos TAG Nisihara R. Direct antiglobulin (Coombs) test in systemic lupus erythematosus patients. Clin Rheumatol. (2017) 36:2141–4. 10.1007/s10067-017-3778-3 28762061

[11] Nandakumar KS. Targeting IgG in arthritis: disease pathways and therapeutic avenues. Int J Mol Sci. (2018) 19:E677. 10.3390/ijms19030677 29495570 PMC5877538

[12] Gibelin A Maldini C Mahr A. Epidemiology and etiology of Wegener granulomatosis, microscopic polyangiitis, Churg-Strauss syndrome and Goodpasture syndrome: vasculitides with frequent lung involvement. Semin Respir Crit Care Med. (2011) 32:264–73. 10.1055/s-0031-1279824 21674413

[13] Hilhorst M van Paassen P Tervaert JW. Proteinase 3-ANCA vasculitis versus myeloperoxidase-ANCA vasculitis. J Am Soc Nephrol. (2015) 26:2314–27. 10.1681/ASN.2014090903 25956510 PMC4587702

[14] Grassegger A Pohla-Gubo G Frauscher M Hintner H. Autoantibodies in systemic sclerosis (scleroderma): clues for clinical evaluation, prognosis and pathogenesis. Wien Med Wochenschr. (2008) 158:19–28. 10.1007/s10354-007-0451-5 18286246

[15] Baroni SS Santillo M Bevilacqua F Luchetti M Spadoni T Mancini M. Stimulatory autoantibodies to the PDGF receptor in systemic sclerosis. N Engl J Med. (2006) 354:2667–76. 10.1056/NEJMoa052955 16790699

[16] Kill A Riemekasten G. Functional autoantibodies in systemic sclerosis pathogenesis. Curr Rheumatol Rep. (2015) 17:34. 10.1007/s11926-015-0505-4 25876754

[17] Rudolph EH Congdon KL Sackey FN Fitzsimons MM Foster MH. Humoral autoimmunity to basement membrane antigens is regulated in C57BL/6 and MRL/MpJ mice transgenic for anti-laminin Ig receptors. J Immunol. (2002) 168:5943–53. 10.4049/jimmunol.168.11.5943 12023401

[18] Brady GF Congdon KL Clark AG Sackey FN Rudolph EH Radic MZ. Kappa editing rescues autoreactive B cells destined for deletion in mice transgenic for a dual specific anti-laminin Ig. J Immunol. (2004) 172:5313–21. 10.4049/jimmunol.172.9.5313 15100270

[19] Clark AG Fan Q Brady GF Mackin KM Coffman ED Weston ML. Regulation of basement membrane-reactive B cells in BXSB, (NZBxNZW)F1, NZB, and MRL/lpr lupus mice. Autoimmunity. (2013) 46:188–204. 10.3109/08916934.2012.746671 23157336 PMC3625511

[20] Clark AG Buckley ES Foster MH. Altered toll-like receptor responsiveness underlies a dominant heritable defect in B cell tolerance in autoimmune New Zealand Black mice. Eur J Immunol. (2018) 48:492–7. 10.1002/eji.201747287 29251774 PMC5937850

[21] O'Sullivan FX Fassbender HG Gay S Koopman WJ. Etiopathogenesis of the rheumatoid arthritis-like disease in MRL/l mice. I. The histomorphologic basis of joint destruction. Arthritis Rheum. (1985) 28:529–36. 10.1002/art.1780280511 4004962

[22] Harper JM Thiru S Lockwood CM Cooke A. Myeloperoxidase autoantibodies distinguish vasculitis mediated by anti-neutrophil cytoplasm antibodies from immune complex disease in MRL/Mp-lpr/lpr mice: a spontaneous model for human microscopic angiitis. Eur J Immunol. (1998) 28:2217–26. 9692891 10.1002/(SICI)1521-4141(199807)28:07<2217::AID-IMMU2217>3.0.CO;2-P

[23] Santiago-Raber ML Baccala R Haraldsson KM Choubey D Stewart TA Kono DH. Type-I interferon receptor deficiency reduces lupus-like disease in NZB mice. J Exp Med. (2003) 197:777–88. 10.1084/jem.20021996 12642605 PMC2193854

[24] DeHeer DH Edgington TS. Evidence for a B lymphocyte defect underlying the anti-X anti-erythrocyte autoantibody response of NZB mice. J Immunol. (1977) 118:1858–63. 323360

[25] Morel L. Genetics of SLE: evidence from mouse models. Nat Rev Rheumatol. (2010) 6:348–57. 10.1038/nrrheum.2010.63 20440287

[26] Rogers NJ Lees MJ Gabriel L Maniati E Rose SJ Potter PK. A defect in Marco expression contributes to systemic lupus erythematosus development via failure to clear apoptotic cells. J Immunol. (2009) 182:1982–90. 10.4049/jimmunol.0801320 19201851

[27] Santiago-Raber M-L Kikuchi S Borel P Uematsu S Akira S Kotzin BL. Evidence for genes in addition to Tlr7 in the Yaa translocation linked with acceleration of systemic lupus erythematosus. J Immunol. (2008) 181:1556–62. 10.4049/jimmunol.181.2.1556 18606711

[28] Fitzsimons MM Chen H Foster MH. Diverse endogenous light chains contribute to basement membrane reactivity in nonautoimmune mice transgenic for an anti-laminin Ig heavy chain. Immunogenetics. (2000) 51:20–9. 10.1007/s002510050004 10663558

[29] Ben-Yehuda A Rasooly L Bar-Tana R Breuer G Tadmor B Ulmansky R. The urine of SLE patients contains antibodies that bind to the laminin component of the extracellular matrix. J Autoimmun. (1995) 8:279–91. 10.1006/jaut.1995.0021 7612153

[30] Amital H Heilweil M Ulmansky R Szafer F Bar-Tana R Morel L. Treatment with a laminin-derived peptide suppresses lupus nephritis. J Immunol. (2005) 175:5516–23. 10.4049/jimmunol.175.8.5516 16210660

[31] Groth S Vafia K Recke A Dahnrich C Zillikens D Stocker W. Antibodies to the C-terminus of laminin gamma1 are present in a distinct subgroup of patients with systemic and cutaneous lupus erythematosus. Lupus. (2012) 21:1482–3. 10.1177/0961203312460113 22968451

[32] Inagaki J Kondo A Lopez LR Shoenfeld Y Matsuura E. Pregnancy loss and endometriosis: pathogenic role of anti-laminin-1 autoantibodies. Ann N Y Acad Sci. (2005) 1051:174–84. 10.1196/annals.1361.059 16126957

[33] Wolff PG Kuhl U Schultheiss HP. Laminin distribution and autoantibodies to laminin in dilated cardiomyopathy and myocarditis. Am Heart J. (1989) 117:1303–9. 10.1016/0002-8703(89)90410-9 2658521

[34] Foster MH Basement membranes and autoimmune diseases. Matrix Biol. (2017) 57–58:149–68. 10.1016/j.matbio.2016.07.008 PMC5290253 27496347

[35] Ghio AJ Jaskot RH Hatch GE. Lung injury after silica instillation is associated with an accumulation of iron in rats. Am J Physiol. (1994) 267:L686–92. 10.1152/ajplung.1994.267.6.L686 7810673

[36] Ghio AJ Tong H Soukup JM Dailey LA Cheng WY Samet JM. Sequestration of mitochondrial iron by silica particle initiates a biological effect. Am J Physiol Lung Cell Mol Physiol. (2013) 305:L712–24. 10.1152/ajplung.00099.2013 23997175

[37] Huaux F Louahed J Hudspith B Meredith C Delos M Renauld JC. Role of interleukin-10 in the lung response to silica in mice. Am J Respir Cell Mol Biol. (1998) 18:51–9. 10.1165/ajrcmb.18.1.2911 9448045

[38] Barbarin V Nihoul A Misson P Arras M Delos M Leclercq I. The role of pro- and anti-inflammatory responses in silica-induced lung fibrosis. Respir Res. (2005) 6:112. 10.1186/1465-9921-6-112 16212659 PMC1274346

[39] Mayeux JM Escalante GM Christy JM Pawar RD Kono DH Pollard KM. Silicosis and silica-induced autoimmunity in the diversity outbred mouse. Front Immunol. (2018) 9:874. 10.3389/fimmu.2018.00874 29755467 PMC5932595

[40] Tighe RM Birukova A Yaeger MJ Reece SW Gowdy KM. Euthanasia- and lavage-mediated effects on bronchoalveolar measures of lung injury and inflammation. Am J Respir Cell Mol Biol. (2018) 59:257–66. 10.1165/rcmb.2017-0357OC 29481287 PMC6096344

[41] Pinchai N Perfect BZ Juvvadi PR Fortwendel JR Cramer RA Jr Asfaw YG. Aspergillus fumigatus calcipressin CbpA is involved in hyphal growth and calcium homeostasis. Eukaryot Cell. (2009) 8:511–9. 10.1128/EC.00336-08 19252123 PMC2669200

[42] McDonald JW Roggli VL. Detection of silica particles in lung tissue by polarizing light microscopy. Arch Pathol Lab Med. (1995) 119:242–6. 7887777

[43] Madaio M Hodder S Schwartz R Stollar B. Responsiveness of autoimmune and normal mice to nucleic acid antigens. J Immunol. (1984) 132:872. 6690621

[44] Ali R Dersimonian H Stollar BD. Binding of monoclonal anti-native DNA autoantibodies to DNA of varying size and conformation. Mol Immunol. (1985) 22:1415–22. 10.1016/0161-5890(85)90065-3 3879530

[45] Foster MH Sabbaga J Line SRP Thompson KS Barrett KJ Madaio MP Molecular analysis of nephrotropic anti-laminin antibodies from an MRL/lpr autoimmune mouse. J Immunol. (1993) 151:814–24. 8335911

[46] Clark AG Weston ML Foster MH. Lack of galectin-1 or galectin-3 alters B cell deletion and anergy in an autoantibody transgene model. Glycobiology. (2013) 23:893–903. 10.1093/glycob/cwt026 23550149 PMC3671777

[47] Cascalho M Ma A Lee S Masat L Wabl M. A quasi-monoclonal mouse. Science. (1996) 272:1649–52. 10.1126/science.272.5268.1649 8658139

[48] Rui L Vinuesa C Blasioli J Goodnow C. Resistance to CpG DNA-induced autoimmunity through tolerogenic B cell antigen receptor ERK signaling. Nat Immunol. (2003) 4:594–600. 10.1038/ni924 12740574

[49] Rui L Healy JI Blasioli J Goodnow CC. ERK signaling is a molecular switch integrating opposing inputs from B cell receptor and T cell cytokines to control TLR4-driven plasma cell differentiation. J Immunol. (2006) 177:5337–46. 10.4049/jimmunol.177.8.5337 17015719

[50] O'Neill SK Veselits ML Zhang M Labno C Cao Y Finnegan A. Endocytic sequestration of the B cell antigen receptor and toll-like receptor 9 in anergic cells. Proc Natl Acad Sci USA. (2009) 106:6262–7. 10.1073/pnas.0812922106 19332776 PMC2662959

[51] Lee SR Rutan JA Monteith AJ Jones SZ Kang SA Krum KN. Receptor cross-talk spatially restricts p-ERK during TLR4 stimulation of autoreactive B cells. J Immunol. (2012) 189:3859–68. 10.4049/jimmunol.1200940 22984080 PMC3466401

[52] Teodorovic LS Babolin C Rowland SL Greaves SA Baldwin DP Torres RM. Activation of Ras overcomes B-cell tolerance to promote differentiation of autoreactive B cells and production of autoantibodies. Proc Natl Acad Sci USA. (2014) 111:E2797–806. 10.1073/pnas.1402159111 24958853 PMC4103347

[53] Yasuda T Kometani K Takahashi N Imai Y Aiba Y Kurosaki T. ERKs induce expression of the transcriptional repressor Blimp-1 and subsequent plasma cell differentiation. Sci Signal. (2011) 4:ra25. 10.1126/scisignal.2001592 21505187

[54] Tomaru M Matsuoka M. The role of mitogen-activated protein kinases in crystalline silica-induced cyclooxygenase-2 expression in A549 human lung epithelial cells. Toxicol Mech Methods. (2011) 21:513–9. 10.3109/15376516.2011.568982 21470077

[55] Yu L Wang L Chen S. Endogenous toll-like receptor ligands and their biological significance. J Cellular and Mol Med. (2010) 14:2592–603. 10.1111/j.1582-4934.2010.01127.x 20629986 PMC4373479

[56] Ntoufa S Papakonstantinou N Apollonio B Gounari M Galigalidou C Fonte E B cell anergy modulated by TLR1/2 and the miR-17 approximately 92 cluster underlies the indolent clinical course of chronic lymphocytic leukemia stereotyped subset #4. J Immunol. (2016) 196:4410–7. 10.4049/jimmunol.1502297 27059597

[57] Pietras EM. Inflammation: a key regulator of hematopoietic stem cell fate in health and disease. Blood. (2017) 130:1693–8. 10.1182/blood-2017-06-780882 28874349 PMC5639485

[58] Bates MA Brandenberger C Langohr I Kumagai K Harkema JR Holian A. Silica triggers inflammation and ectopic lymphoid neogenesis in the lungs in parallel with accelerated onset of systemic autoimmunity and glomerulonephritis in the lupus-prone NZBWF1 mouse. PLoS ONE. (2015) 10:e0125481. 10.1371/journal.pone.0125481 25978333 PMC4433215

[59] Kilmon MA Rutan JA Clarke SH Vilen BJ. Low-affinity, Smith antigen-specific B cells are tolerized by dendritic cells and macrophages. J Immunol. (2005) 175:37–41. 10.4049/jimmunol.175.1.37 15972629 PMC3724409

[60] Kilmon MA Wagner NJ Garland AL Lin L Aviszus K Wysocki LJ. Macrophages prevent the differentiation of autoreactive B cells by secreting CD40 ligand and interleukin-6. Blood. (2007) 110:1595–602. 10.1182/blood-2006-12-061648 17712049 PMC1952615

[61] Gilbert MR Wagner NJ Jones SZ Wisz AB Roques JR Krum KN. Autoreactive preplasma cells break tolerance in the absence of regulation by dendritic cells and macrophages. J Immunol. (2012) 189:711–20. 10.4049/jimmunol.1102973 22675201 PMC3392546

[62] Vanhee D Gosset P Boitelle A Wallaert B Tonnel AB. Cytokines and cytokine network in silicosis and coal workers' pneumoconiosis. Eur Respir J. (1995) 8:834–42. 7656959

[63] Davis GS Leslie KO Hemenway DR. Silicosis in mice: effects of dose, time, and genetic strain. J Environ Pathol Toxicol Oncol. (1998) 17:81–97. 9546745

[64] Brown JM Archer AJ Pfau JC Holian A. Silica accelerated systemic autoimmune disease in lupus-prone New Zealand mixed mice. Clin Exp Immunol. (2003) 131:415–21. 10.1046/j.1365-2249.2003.02094.x 12605693 PMC1808650

[65] Bates MA Akbari P Gilley KN Wagner JG Li N Kopec AK. Dietary docosahexaenoic acid prevents silica-induced development of pulmonary ectopic germinal centers and glomerulonephritis in the lupus-prone NZBWF1 mouse. Front Immunol. (2018) 9:2002. 10.3389/fimmu.2018.02002 30258439 PMC6143671

[66] Moyron-Quiroz JE Rangel-Moreno J Hartson L Kusser K Tighe MP Klonowski KD. Persistence and responsiveness of immunologic memory in the absence of secondary lymphoid organs. Immunity. (2006) 25:643–54. 10.1016/j.immuni.2006.08.022 17045819

[67] Maglione PJ Xu J Chan J. B cells moderate inflammatory progression and enhance bacterial containment upon pulmonary challenge with *Mycobacterium tuberculosis*. J Immunol. (2007) 178:7222–34. 10.4049/jimmunol.178.11.7222 17513771

[68] GeurtsvanKessel CH Willart MA Bergen IM van Rijt LS Muskens F Elewaut D. Dendritic cells are crucial for maintenance of tertiary lymphoid structures in the lung of influenza virus-infected mice. J Exp Med. (2009) 206:2339–49. 10.1084/jem.20090410 19808255 PMC2768850

[69] Randall TD. Bronchus-associated lymphoid tissue (BALT) structure and function. Adv Immunol. (2010) 107:187–241. 10.1016/B978-0-12-381300-8.00007-1 21034975 PMC7150010

[70] Le Pottier L Devauchelle V Fautrel A Daridon C Saraux A Youinou P Ectopic germinal centers are rare in Sjogren's syndrome salivary glands and do not exclude autoreactive B cells. J Immunol. (2009) 182:3540–7. 10.4049/jimmunol.0803588 19265132

[71] Croia C Serafini B Bombardieri M Kelly S Humby F Severa M. Epstein-Barr virus persistence and infection of autoreactive plasma cells in synovial lymphoid structures in rheumatoid arthritis. Ann Rheum Dis. (2013) 72:1559–68. 10.1136/annrheumdis-2012-202352 23268369

[72] Corsiero E Bombardieri M Carlotti E Pratesi F Robinson W Migliorini P. Single cell cloning and recombinant monoclonal antibodies generation from RA synovial B cells reveal frequent targeting of citrullinated histones of NETs. Ann Rheum Dis. (2016) 75:1866–75. 10.1136/annrheumdis-2015-208356 26659717 PMC5036240

[73] Kinjoh K Kyogoku M Good R. Genetic selection for crescent formation yields mouse strain with rapidly progressive glomerulonephritis and small vessel vasculitis. Proc Natl Acad Sci USA. (1993) 90:3413–7. 10.1073/pnas.90.8.3413 8475090 PMC46310

[74] Neumann I Birck R Newman M Schnulle P Kriz W Nemoto K. SCG/Kinjoh mice: a model of ANCA-associated crescentic glomerulonephritis with immune deposits. Kidney Int. (2003) 64:140–8. 10.1046/j.1523-1755.2003.00061.x 12787404

[75] Xiao H Heeringa P Hu P Liu Z Zhao M Aratani Y. Antineutrophil cytoplasmic autoantibodies specific for myeloperoxidase cause glomerulonephritis and vasculitis in mice. J Clin Invest. (2002) 110:955–63. 10.1172/JCI200215918 12370273 PMC151154

[76] Alsughayyir J Pettigrew GJ Motallebzadeh R. Spoiling for a fight: B lymphocytes as initiator and effector populations within tertiary lymphoid organs in autoimmunity and transplantation. Front Immunol. (2017) 8:1639. 10.3389/fimmu.2017.01639 29218052 PMC5703719

