# Supplementary material for: Silica Exposure Differentially Modulates Autoimmunity in Lupus Strains and Autoantibody Transgenic Mice
Source: Front Immunol. 2019 Oct 1;10:2336. doi: 10.3389/fimmu.2019.02336 (PMC6781616; doi:10.3389/fimmu.2019.02336)
Supplement: Supplementary file 1 [file Image_1.pdf]

## **Figure** S.1.

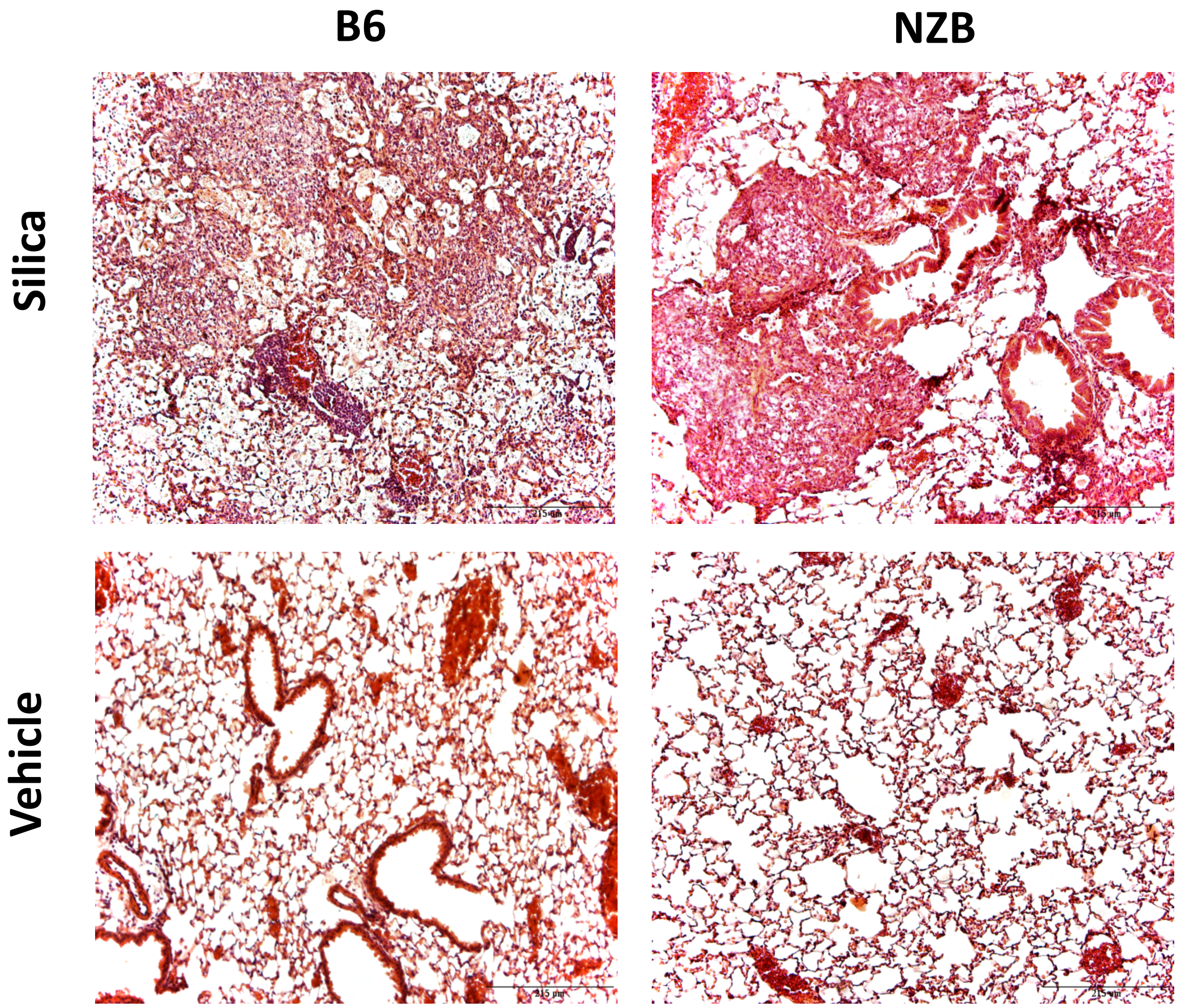


Lung injury and inflammation from mice one month after exposure.
Representative sections from mice of indicated strain; H&E, original magnification x40.
