# Supplementary material for: Silica Exposure Differentially Modulates Autoimmunity in Lupus Strains and Autoantibody Transgenic Mice
Source: Front Immunol. 2019 Oct 1;10:2336. doi: 10.3389/fimmu.2019.02336 (PMC6781616; doi:10.3389/fimmu.2019.02336)
Supplement: Supplementary file 2 [file Image_2.pdf]

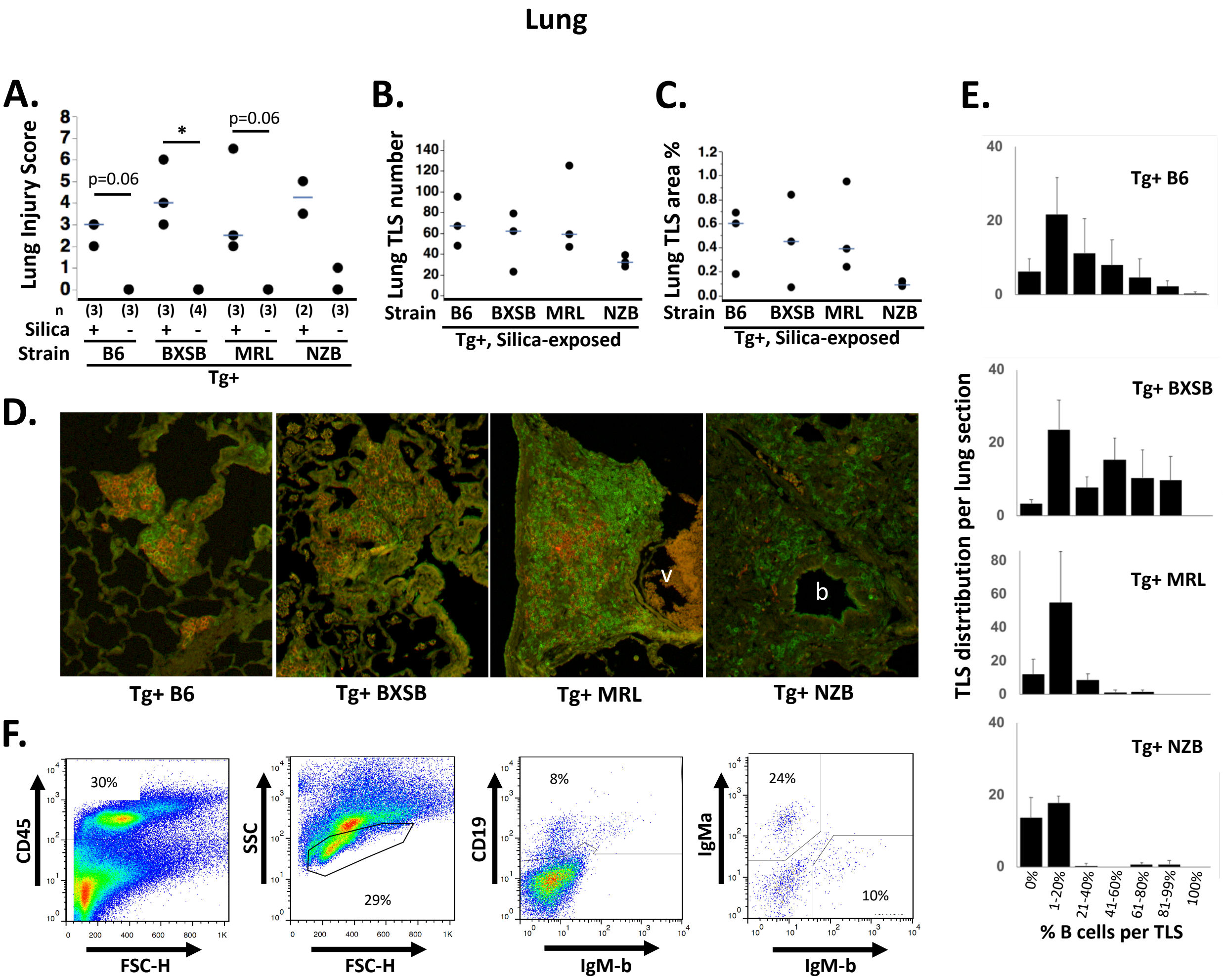

Lung injury and tertiary lymphoid structures (TLS) and lung cell flow cytometry gating strategy in autoantibody transgenic (autoAb Tg) mice after exposure to crystalline silica or vehicle. (A) Lung injury composite score, (B) TLS number per whole lung section, and (C) TLS area as percentage of area of whole lung section, by strain and exposure; * p<0.05. (D) Representative sections of lung from autoAb Tg mice of indicated strain 1.75 (B6 and BXSB), 1.5 (MRL), and 0.9 (NZB) months after instillation of silica, stained with anti-B220 (B cells, red) and anti-CD3e (T cells, green). (E) Semiquantitative assessment of proportion of B cells per TLS. Each TLS was scored as 0%, 1-20%, 21-40%, 41-60%, 61-80%, 81-99%, or 100% B cells; bars show number of TLS in each category as mean±SD, by strain. (F) Representative flow cytometry from isolated lung cells from a silica-exposed NZB mouse. Gating strategy is as shown. IgMa and IgMb indicate Tg and endogenous IgM B cells, respectively.

Fig. S.2
