# Supplementary material for: Silica Exposure Differentially Modulates Autoimmunity in Lupus Strains and Autoantibody Transgenic Mice
Source: Front Immunol. 2019 Oct 1;10:2336. doi: 10.3389/fimmu.2019.02336 (PMC6781616; doi:10.3389/fimmu.2019.02336)
Supplement: Supplementary file 3 [file Image_3.pdf]

Spleen

A. **B.**

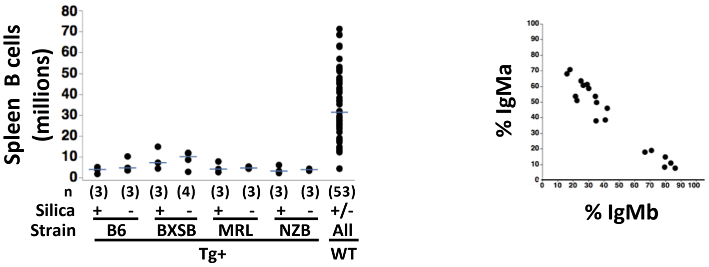

(A) Spleen CD19+ B cell numbers as determined by flow cytometric analysis in silica- or vehicle-exposed autoAb Tg mice. Strains are indicated; for comparison to B cell numbers in wildtype (non-autoAb Tg) mice, spleen B cell counts from wildtype mice described in this paper are pooled (All, n=53). Each symbol represents an individual mouse; the median for each group is indicated by the bar. (B) Relative expression of autoAb Tg IgMa versus endogenous IgMb on splenic B cells, as determined by flow cytometry, for B6, BXSB and NZB autoAb Tg mice. The 6 B6 subjects are represented in the cluster of symbols with low % IgMa and high % IgMb in the lower right section of graph. Each symbol represents an individual mouse. (MRL mice express endogenous IgMj allotype that crossreacts with IgMa; samples from MRL autoAb Tg subjects are not included in this assay.)

Fig. S.3
