# Supplementary material for: Silica Exposure Differentially Modulates Autoimmunity in Lupus Strains and Autoantibody Transgenic Mice
Source: Front Immunol. 2019 Oct 1;10:2336. doi: 10.3389/fimmu.2019.02336 (PMC6781616; doi:10.3389/fimmu.2019.02336)
Supplement: Supplementary file 4 [file Image_4.pdf]

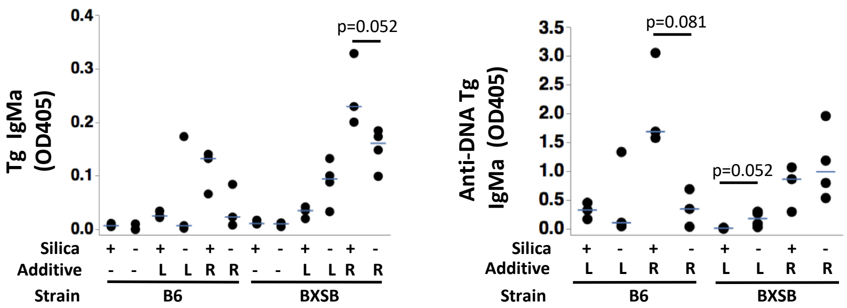

Level of IgMa (Tg) (left) and anti-ssDNA Tg autoAb (right) in supernatants of spleen cells from exposed mice stimulated 7-8 days with indicated additive: -, none; L, lipopolysaccharide (TLR4 ligand); R, R848 (TLR7 ligand) in combination with CpG oligos (TLR9 ligand). Undiluted supernatants were tested in duplicate; n=3 mice/strain. Concurrent OD405 for positive control monoclonal antibody was 3.169 for anti-DNA H241 IgG. For DNA binding results, there was insufficient sample for testing without additive. Each symbol represents an individual mouse; the median for each group is indicated by the bar.

Supplemental Fig S.4
