# Supplementary material for: Silica Exposure Differentially Modulates Autoimmunity in Lupus Strains and Autoantibody Transgenic Mice
Source: Front Immunol. 2019 Oct 1;10:2336. doi: 10.3389/fimmu.2019.02336 (PMC6781616; doi:10.3389/fimmu.2019.02336)
Supplement: Supplementary file 5 [file Table_1.pdf]

Table S.1. Exposure duration and age at harvest for autoAb Tg mice.

Strain	Exposure	Time post-exposure at harvest (wks)	Age at harvest (wks)
B6-Tg	Si	7.3	48.4, 78, 78.3
	V	7.3	43, 48.4, 78.3
BXSB-Tg	Si	7.3	46.4, 51.7, 51.7
	V	7.3	46.4, 50.9, 51.7, 51.7
MRL-Tg	Si	6.3	25.4, 49.1, 56.9
	V	6.3	25.4, 49.1, 56.9
NZB-Tg	Si	3.4 [a]	27.6, 34.6, 45.6
	V	3.4	27.6, 35.1, 35.1

a. NZB-Tg mice were harvested early after weight loss and death occurred in 3 exposed NZB-Tg mice (not included in these analyses).
